# Supplementary material for: Cholesterol-Induced Nanoscale Variations in the Thickness of Phospholipid Membranes
Source: Nano Lett. 2023 Jan 27;23(6):2421–6. doi: 10.1021/acs.nanolett.2c04635 (PMC10037415; doi:10.1021/acs.nanolett.2c04635)
Supplement: Supplementary file 1 — nl2c04635_si_001.pdf [file nl2c04635_si_001.pdf]

Supporting Information: Cholesterol-Induced Nanoscale Variations in the Thickness of Phospholipid Membranes

Tao Chen,† Arindam Ghosh,† and Jörg Enderlein*,†,‡

†*Third Institute of Physics – Biophysics, Georg August University, Friedrich-Hund-Platz 1, 37077 Göttingen, Germany*

‡*Cluster of Excellence "Multiscale Bioimaging: from Molecular Machines to Networks of Excitable Cells" (MBExC), Universitätsmedizin Göttingen, Robert-Koch-Str. 40, 37075 Göttingen, Germany*

E-mail: jenderl@gwdg.de

Reagents

All unlabeled lipids, 1,2-dioleoyl-sn-glycero-3-phosphocholine (DOPC), 1,2-dilauroyl-sn-glycero-3-phosphocholine (DLPC), 1,2-diphytanoyl-sn-glycero-3-phosphocholine (DPhPC) and cholesterol were purchased from Avanti Polar Lipids (Alabama, USA). Atto655-labelled 1,2-dipalmitoyl-sn-glycero-3-phosphoethanolamine (DPPE), 1,2-Dimyristoyl-sn-glycero-3-phospho ethanolamine (DMPE), and 1,2-dilauroyl-sn-glycero-3-phosphoethanolamine (DLPE) were purchased from ATTO-TEC GmbH (Siegen, Germany). All unlabeled lipids were diluted to 10 mg/ml in chloroform whereas the fluorescently labeled lipids were diluted to 0.01 mg/ml for preparation of a stock solution. Cholesterol was diluted to 20 mg/ml in chloroform for preparing a stock solution.

Substrate preparation

Coverslips coated with single sheets of graphene were prepared by a transfer method based on the manufacturer's instruction. Briefly, the purchased monolayer graphene (Easy Transfer Monolayer, Graphenea) was sandwiched between a polymer film and a thin sacrificial film and cut into $\sim$1$\times$1 cm^2 pieces, which were submersed into water for removing the polymer film. The floating graphene sheet with the sacrificial film was captured with a plasma-cleaned coverslip and dried at room temperature for 30 min, followed by heating at 150°C for 1 h. Dried graphene-coated coverslips were stored under vacuum for at least 24 h to prevent detachment of the graphene from the coverslip. Finally, the sacrificial layer was removed by placing the coverslip into hot acetone (50°C) for 1 h and into isopropyl alcohol for another 1 h. Coverslips were dried in a stream of N_2 and stored in a desiccator. In a next step, graphene-coated coverslips were coated with a SiO_2 spacer of 10 nm thickness by chemical vapor deposition using an electron beam source (Univex 350, Leybold) under high vacuum conditions (10^{-5} mbar). Deposition was done at a slow rate of 1 Ås^{-1} to ensure maximal homogeneity of the deposited quartz layer. Layer thickness was continuously monitored during deposition with an oscillating quartz unit.

Sample preparation

Small unilamellar vesicles (SUV) of DLPC and DOPC with and without cholesterol were prepared by extrusion. Briefly, a 60 µl droplet of chloroform solution containing unlabeled lipids (10 mg/ml DLPC or DOPC), cholesterol (15, 30 and 44 mol%), and 1 µl of 0.01 mg/ml DLPE-Atto655 (for DLPC SLB) or DPPE-Atto655 (for DOPC) was dried in vacuum for 1.5 h at 30°C to evaporate the chloroform. The obtained lipid film was re-suspended with 500 µl of Tris buffer (20 mM Tris-Cl, 100 mM NaCl, 10 mM $CaCl_2$, pH 7.4) in an ultra-

sonic bath for 5 min, followed by stirring (Thermomixer Comfort, Eppendorf) at 30°C for 1 h. The solution was then extruded for 15 cycles through a polycarbonate filter (Whatman) with 50 nm pore diameter. The resulting vesicle solutions were used within 3 days and stored at 4°C before use. Giant unilamellar vesicles (GUV) of DPhPC with and without cholesterol were prepared by electro-formation as described before. Briefly, 100 µl of a chloroform solution containing DPhPC lipid (10 mg/ml) and cholesterol (15, 30 and 44 mol%) and 2 µl of 0.01 mg/ml DMPE-Atto655 was filled into a custom-built chamber, followed by evaporation for 3 h under vacuum at 30°C. The chamber was re-filled with 500 µl of 300 mM sucrose solution, after which an alternating electric current of 15 Hz frequency and a peak-to-peak voltage of 1.6 V was applied for 3 h, followed by a lower frequency voltage of 8 Hz for another 30 min. Formed GUVs were collected by rinsing the electrode surface with 500 µl of a Tris-Cl buffer solution. Next, DLPC and DOPC SLBs were formed via vesicle fusion. Before placing a SUV solution onto the GIET substrate, the substrate's surface was activated with a plasma cleaner (Harrick Plasma, New York, United States) at low intensity for 30 s. After that, a droplet of SUV solution was deposited on the substrate and incubated for 3 h to ensure the formation of a uniform bilayer with minimal defects. This was followed by washing with copious buffer. For forming a homogeneous SLB with high cholesterol content, samples were incubated at room temperature for 6 h before further measurements. DPhPC SLBs were formed by putting a droplet of diluted GUV solution (10 times dilution from the stock solution) onto the GIET substrate and incubating for 10 min, followed by washing with buffer solution.

Experimental setup

Fluorescence lifetime measurements were done with a home-built confocal microscope equipped with an objective lens of high numerical aperture (ApoN, 100× oil, 1.49 NA, Olympus Europe). For fluorescence excitation, the light of a pulsed diode laser (λ_{exc} =

640 nm, LDH-D-C 640, PicoQuant GmbH, Berlin, Germany) with 50 ps FWHM pulse width and 40 MHz repetition rate was used. A clean-up filter (Z640/10, Chroma Technology, Bavaria, Germany) was used in the excitation path. Laser light was guided towards the microscope with a polarisation-maintaining single-mode optical fiber (PMC-400-4.2-NA010-3-APC250 V, Schäfter and Kirchhoff, Hamburg, Germany). After the optical fiber, the light was collimated into a parallel light beam of 12 mm diameter with an infinity-corrected 4x objective lens (UPlSApo 4X, Olympus). A quad-band dichroic mirror (Di01-R405/488/561/635, Semrock, New York, United States) reflected this beam towards the objective lens. Fluorescence measurements were done at a total laser power of $\sim$20 µW as measured at the back focal plane of the objective lens. Fluorescence was collected through the same objective (epi-fluorescence excitation/detection). After passing the dichroic mirror, the collected fluorescence light was sent though a long-pass filter (BLP01-635R, Semrock) for blocking back-scattered excitation light, and focused through a confocal pinhole of 100 µm diameter. After the pinhole, the light was re-focused onto the active area of a single-photon avalanche diode (SPCM-AQRH, Excelitas Technologies Corp., Mississauga, Canada) for single photon detection. Photon detection timing was done with a multi-channel picosecond event timer (HydraHarp 400, PicoQuant GmbH).

Determination of dye orientation

To determine the orientation of fluorophore atto655 in the membrane, we prepared two kinds of GUVs: 1) Pure DOPC and DPPE-atto655; 2) 30 mol% Chol in DOPC and DPPE-atto655. Then the GUVs are imaged under polarized excitation light by a epi-fluorescence microscopy. As shown in Figure S1 (3), both for the two GUVs, the observed intensity distribtuion is consistent with a dye orientation parallel to the bilayer surface.

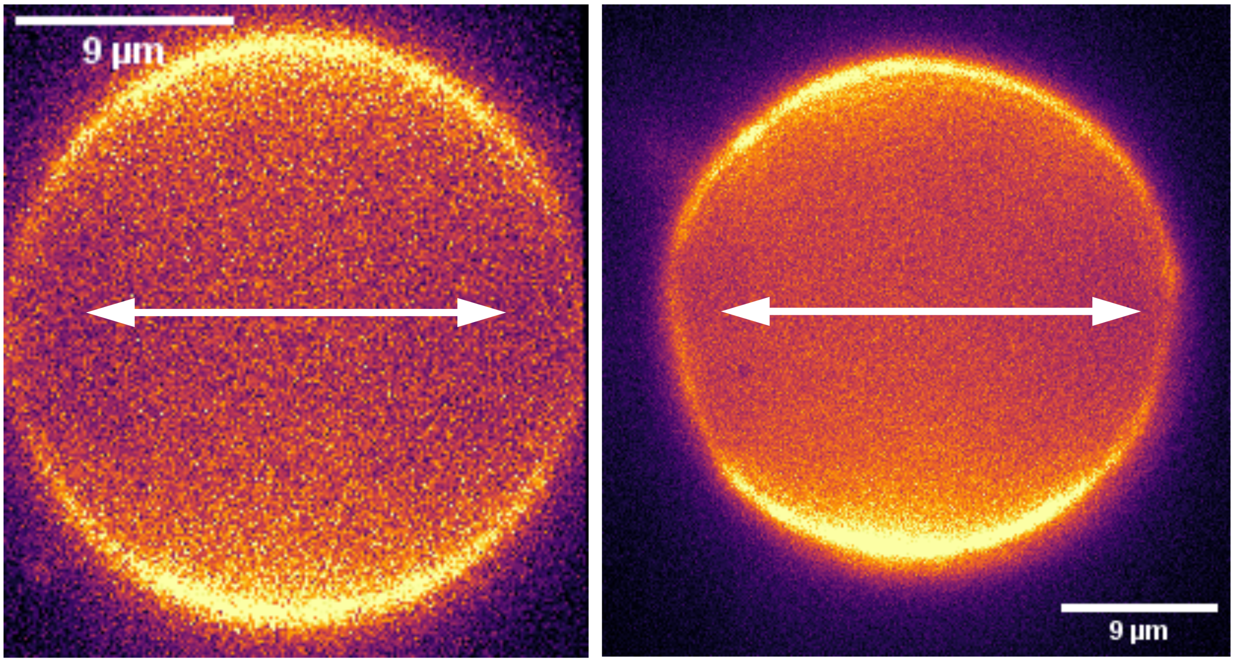

Figure S 1: **Orientation of the Atto655.** Images of GUV made of pure DOPC and DPPE-atto655 (left) and GUV made of DOPC, 30 mol% Chol, and DPPE-atto655 (right) under linearly polarized excitation (the double-headed arrow indicates the excitation polarization).

Working principle of GIET

The inset of Figure 1(A) in the main text shows the geometry of a GIET substrate with a SLB on top. The GIET substrate itself consists of a single sheet of graphene sandwiched between a glass coverslip and a thin quartz layer (10 nm of SiO_2). The SLB on top contains fluorescently head-group labeled lipids. Excitation and detection of fluorophores is done from the bottom through the substrate. The theory of GIET has been explained elsewhere.[1] Briefly, graphene acts as an efficient energy acceptor of the excited state energy of a fluorophore, similar to an acceptor dye in classical Förster resonance energy transfer. This leads to a strongly distance-dependent modulation of fluorescence intensity and excited-state lifetime of the fluorophore over a distance range of $\sim$30 nm above the graphene layer. This modulation can be calculated by modeling the emitting fluorescent molecule as a an electric dipole emitter and solving Maxwell's equation with

this source field in the presence of the graphene substrate. Here, the graphene layer is treated as an ideal homogeneous layer of 3.4 Ångstrom thickness having a (wavelength-dependent) complex-valued refractive index (see Figure 1(B) in main text). By solving Maxwell's equations, one find the emission power of the dipole emitter $S(\theta, z_0)$ as a function of its distance z_0 from the substrate surface and of its relative orientation defined by the angle θ between its dipole axis and the normal to the substrate surface. This emission power is inversely proportional to the radiative transition rate from the electronic excited state to the ground state. Taking into account also the non-radiative transition rate, the observable excited-state fluorescence lifetime (τ_f) is then found as

$$\frac{\tau_f(\theta, z_0)}{\tau_0} = \frac{S_0}{\phi S(\theta, z_0) + (1-\phi)S_0} \tag{1}$$

where τ_0 is the free-space fluorescence lifetime in absence of the graphene layer, ϕ represents the quantum yield of fluorescence of the used fluorophore, and S_0 is the free-space emission power of an ideal electric dipole emitter given by $S_0 = cn{k_0}^4p^2/3$ with c being the speed of light, k_0 the wave vector amplitude in vacuum, n the refractive index of water, and p the amplitude of emission dipole moment vector. GIET exploits this lifetime-on-distance (τ_f versus z_0) dependence for converting measured lifetime values into distance values, see Figure 1 in main text. To calculate this model curve, *a priori* knowledge of the fluorophore's quantum yield (ϕ), free-space lifetime (τ_0) and dipole orientation (θ) is required. Previously, we have determined these values for Atto655 labeled phosphatidylcholine (PC) lipid chains and found the following values: τ_0 = 2.6 ns, ϕ = 0.36, and a fluorophore orientation parallel to the membrane.[1] Using these values, a graphene layer thickness of 0.34 nm and a refractive index of $n_{\text{graphene}} = 2.77 + 1.41i$ (corresponding to an emission wavelength of 680 nm), a quartz layer thickness of 10 nm with refractive index $n_{SiO_2} = 1.46$, and a refractive index of $n_{\mathrm{H_2O}} = 1.33$ for the aqueous solution above the substrate, we calculated the model GIET curve as shown in Figure 1 in the main text. For

the GIET model curve calculation, we also took into account the presence of a 5 nm thick SLB with a refractive index of 1.46.

Data evaluation

Acquired fluorescence lifetime data were chopped into bunches of 10^6 photons, and for each bunch a TCSPC histogram was calculated and fitted with a bi-exponential decay curve convoluted with the instrument response function (IRF) (as described earlier[1]) that was obtained from background-only signal. We used a Nelder-Mead simplex algorithm for minimizing a negative log-likelihood function, with the fluorescence decay lifetimes, their amplitudes, and an IRF color shift as the fit parameters. For conversion of fluorescence lifetimes into distance values, we used the calculated lifetime-versus-distance GIET curve as described above (see also Figure 1 in main text). For this purpose, a custom-written MATLAB script was used. A MATLAB-based software package for the calculation of MIET lifetime-versus-distance curves as well as the conversion of lifetime to distance, equipped with a graphical user interface, has been published[2] and is available free of charge at https://projects.gwdg.de/projects/miet. While the published version of the software assumes that the fluorescent emitters are rotating quickly compared to their excited-state lifetime, this was not the case for the measurements in the present work. Here, a dye orientation parallel to the bilayer (and thus to the substrate) was assumed when calculating the GIET calibration curve.

TCSPC plots and fits

DOPC thickness Comparison

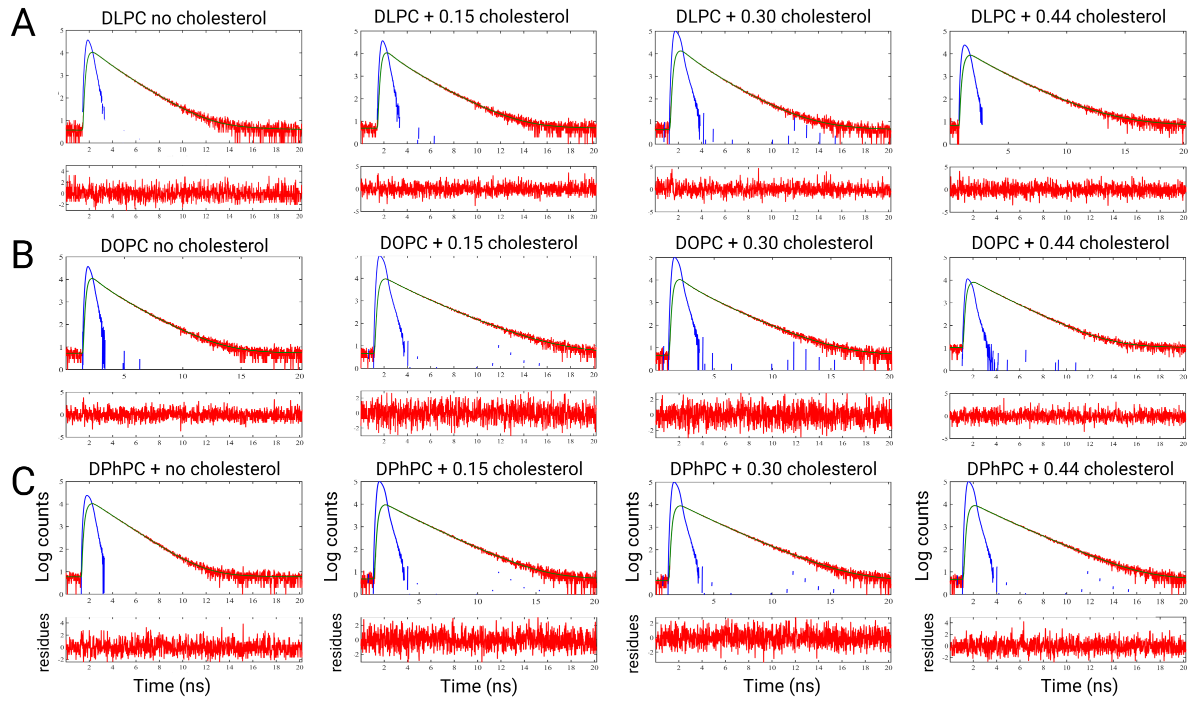

Figure S 2: **TCSPC histograms and bi-exponential fits for representative photon bunches from each lipid type containing 0, 0.15, 0.30, and 0.44 molar fractions of Cholesterol.** Fit residuals are also shown. Row **A.** DLPC **B.** DOPC, and row **C.** DPhPC.

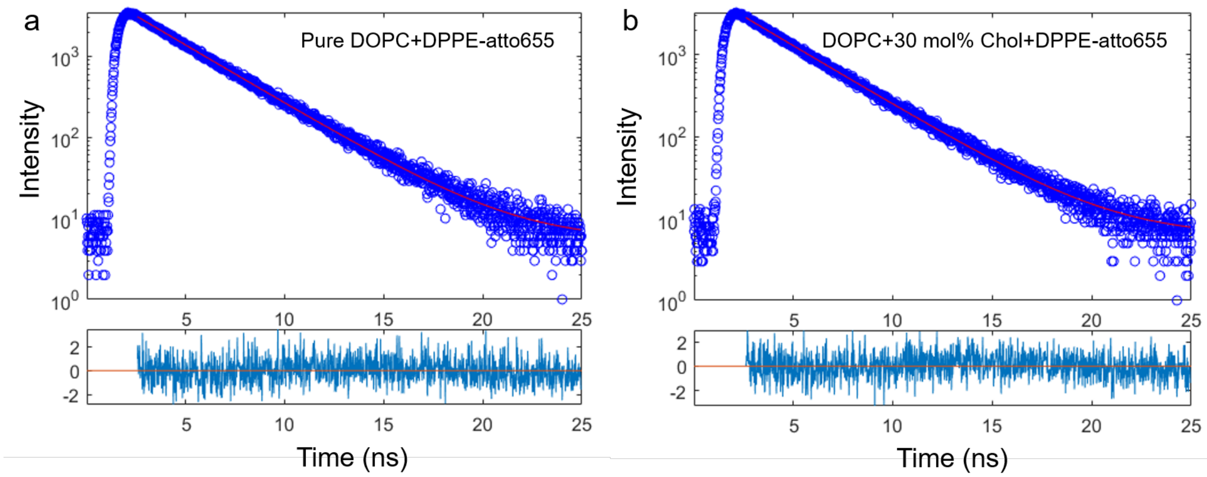

Figure S 3: **A**. TCSPC histogram and fit for Atto655 in DOPC SLB. **B**. TCSPC histogram and fit for Atto655 in DOPC SLB containing 0.30 molar fraction of cholesterol. Both decays are fitted with single exponential confirming the origin of biexponential behavior only in case of GIET experiments where each lifetime value correspond to fluorophores in respective leaflets of the SLB.

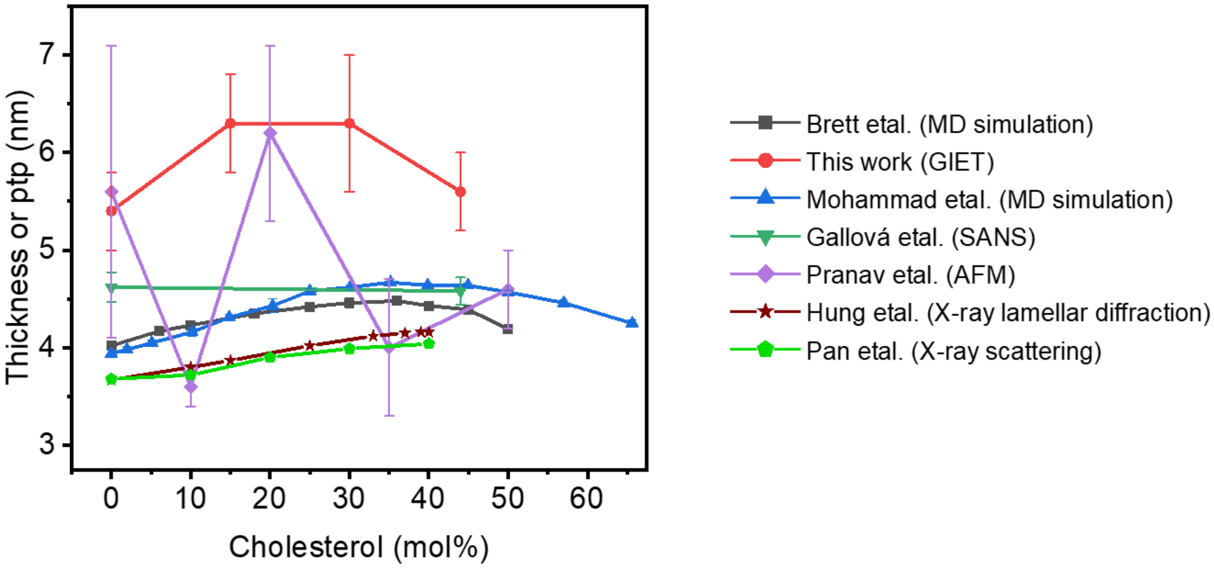

Figure S 4: **T**hicknesses or phosphate-to-phosphate distances (ptp) comparison for DOPC (multiply)-bilayers with different cholesterol fractions obtained from different methods. Data are adapted from published results.[3? –6]

Table S1

DLPC lifetimes and heights				
mol fraction Chol	bottom leaflet lifetime (ns)	top leaflet lifetime (ns)	bottom leaflet height (nm)	top leaflet height (nm)
0	0.88 ± 0.03	1.49 ± 0.07	1.3 ± 0.2	5.0 ± 0.6
0.15	0.99 ± 0.04	1.74 ± 0.02	1.8 ± 0.2	6.7 ± 0.2
0.30	1.01 ± 0.05	1.78 ± 0.04	1.9 ± 0.2	7.0 ± 0.3
0.44	1.01 ± 0.06	1.81 ± 0.03	1.9 ± 0.3	7.3 ± 0.3

DOPC lifetimes and heights				
Chol fraction	bottom leaflet lifetime (ns)	top leaflet lifetime (ns)	bottom leaflet height (nm)	top leaflet height (nm)
0	1.03 ± 0.04	1.83 ± 0.03	2.0 ± 0.2	7.4 ± 0.2
0.15	0.97 ± 0.09	1.89 ± 0.03	1.7 ± 0.5	7.9 ± 0.3
0.30	0.97 ± 0.09	1.90 ± 0.07	1.7 ± 0.5	8.1 ± 0.7
0.44	0.98 ± 0.09	1.82 ± 0.04	1.8 ± 0.5	7.4 ± 0.3

DPhPC lifetimes and heights				
Chol fraction	bottom leaflet lifetime (ns)	top leaflet lifetime (ns)	bottom leaflet height (nm)	top leaflet height (nm)
0	1.26 ± 0.01	1.85 ± 0.05	3.29 ± 0.05	7.6 ± 0.4
0.15	1.2 ± 0.1	1.88 ± 0.05	2.7 ± 0.5	7.9 ± 0.4
0.30	1.09 ± 0.07	1.96 ± 0.06	2.3 ± 0.4	8.7 ± 0.6
0.44	1.36 ± 0.03	2.07 ± 0.07	3.8 ± 0.2	10 ± 1

References

(1) Ghosh, A.; Sharma, A.; Chizhik, A. I.; Isbaner, S.; Ruhlandt, D.; Tsukanov, R.; Gregor, I.; Karedla, N.; Enderlein, J. Graphene-based metal-induced energy transfer for sub-nanometre optical localization. *Nature Photonics* **2019**, *13*, 860–865.

(2) Ghosh, A.; Chizhik, A. I.; Karedla, N.; Enderlein, J. Graphene-and metal-induced energy transfer for single-molecule imaging and live-cell nanoscopy with (sub)-nanometer axial resolution. *Nature Protocols* **2021**, 1–21.

(3) Alwarawrah, M.; Dai, J.; Huang, J. A molecular view of the cholesterol condensing effect in DOPC lipid bilayers. *The Journal of Physical Chemistry B* **2010**, *114*, 7516–7523.

(4) Adhyapak, P. R.; Panchal, S. V.; Murthy, A. V. R. Cholesterol induced asymmetry in DOPC bilayers probed by AFM force spectroscopy. *Biochimica et Biophysica Acta (BBA)-Biomembranes* **2018**, *1860*, 953–959.

(5) Hung, W.-C.; Lee, M.-T.; Chen, F.-Y.; Huang, H. W. The condensing effect of cholesterol in lipid bilayers. *Biophysical Journal* **2007**, *92*, 3960–3967.

(6) Pan, J.; Mills, T. T.; Tristram-Nagle, S.; Nagle, J. F. Cholesterol perturbs lipid bilayers nonuniversally. *Physical Review Letters* **2008**, *100*, 198103.
